# Mercury Contamination in Riverine Sediments and Fish Associated with Artisanal and Small-Scale Gold Mining in Madre de Dios, Peru

**DOI:** 10.3390/ijerph15081584

**Published:** 2018-07-26

**Authors:** Gerardo Martinez, Stephen A. McCord, Charles T. Driscoll, Svetoslava Todorova, Steven Wu, Julio F. Araújo, Claudia M. Vega, Luis E. Fernandez

**Affiliations:** 1 Department of Civil and Environmental Engineering, University of California, Davis, CA 95616, USA; sam@mccenv.com; 2 Department of Civil and Environmental Engineering, Syracuse University College of Engineering and Computer Science, Syracuse, NY 13244, USA; ctdrisco@syr.edu (C.T.D.); stodorov@syr.edu (S.T.); 3 BioConsortia Inc., Davis, CA 95616, USA; stevenhwu@gmail.com; 4 Centro de Innovación Científica Amazónica (CINCIA), Puerto Maldonado, Madre de Dios 17000, Peru; araujojm@wfu.edu (J.F.A.); vegacm@wfu.edu (C.M.V.); fernanle@wfu.edu (L.E.F.); 5 Center for Energy, Environmental and Sustainability (CEES), Wake Forest University, Winston-Salem, NC 27109, USA

**Keywords:** mercury, fish, sediment, artisanal and small-scale gold mining, Madre de Dios, Peruvian Amazon

## Abstract

Artisanal and small-scale gold mining (ASGM) in Madre de Dios, Peru, continues to expand rapidly, raising concerns about increases in loading of mercury (Hg) to the environment. We measured physicochemical parameters in water and sampled and analyzed sediments and fish from multiple sites along one ASGM-impacted river and two unimpacted rivers in the region to examine whether Hg concentrations were elevated and possibly related to ASGM activity. We also analyzed the 308 fish samples, representing 36 species, for stable isotopes (δ^15^N and δ^13^C) to estimate their trophic position. Trophic position was positively correlated with the log-transformed Hg concentrations in fish among all sites. There was a lack of relationship between Hg concentrations in fish and either Hg concentrations in sediments or ASGM activity among sites, suggesting that fish Hg concentrations alone is not an ideal bioindicator of site-specific Hg contamination in the region. Fish Hg concentrations were not elevated in the ASGM-impacted river relative to the other two rivers; however, sediment Hg concentrations were highest in the ASGM-impacted river. Degraded habitat conditions and commensurate shifts in fish species and ecological processes may influence Hg bioaccumulation in the ASGM-impacted river. More research is needed on food web dynamics in the region to elucidate any effects caused by ASGM, especially through feeding relationships and food sources.

## 1. Introduction

Mercury (Hg) is a global pollutant that can affect both human and ecosystem health. It is a naturally-occurring element, but concentrations have been enriched due to mobilization by humans for thousands of years into the atmosphere, as well as aquatic and terrestrial ecosystems [1,2]. Over the past century, Hg emissions have increased due to increased industrialization associated with fossil fuel combustion, mining, and industrial products and processes [2,3].

Artisanal and small-scale gold mining (ASGM) currently accounts for an estimated 37% of global Hg emissions into the atmosphere [4,5]. Mercury-laden waste is also directly discharged into water bodies after gold refining processes. Mercury exposure can threaten wildlife and human health through the consumption of Hg-contaminated fish [6,7,8]. Thus, remote regions and communities near gold mining activities, such as the Amazonian region of Madre de Dios (MDD) in Peru, are highly susceptible to Hg contamination [9,10].

Peru is Latin America’s top gold producer, and currently the sixth largest gold producer globally, processing approximately 150 Mg in 2017 [11,12,13]. ASGM has been occurring in MDD for over 40 years [14]. From 1999 to 2012, land conversion for gold mining in MDD increased 400%, with approximately 50,000 ha of forest removed by 2012 [15]. From 2012 to 2016, another 20,000 ha was deforested due to gold mining in MDD [16]. Asner et al. [15,16] indicate that coinciding with the rapid expansion of mining zones there is also a marked increase in the number of miners. It is estimated that there are over 60,000 miners working in this region [17,18].

Peru signed the United Nations’ Minamata Convention in October 2013 and ratified it in January 2016. The Minamata Convention is a global treaty to limit Hg emissions and releases to the environment and protect human and environmental health from adverse effects of Hg. Despite this international management effort, extrajudicial gold mining continues to expand in Peru. ASG miners mix liquid elemental Hg with river sediments or soils to capture and amalgamate fine alluvial gold particles. The gold-mercury amalgam is heated in an open flame to volatilize the Hg and extract the gold [19,20]. A similar practice of adding liquid Hg to mined sediment slurries was practiced in California during the ‘Gold Rush’ (latter half of 1800s), leaving a legacy of Hg contamination that continues to plague the environment [21].

The toxicity and exposure of Hg is closely linked to the formation of methylmercury (MeHg). Ionic Hg can be converted by bacteria and archaea in anaerobic waters and sediments into the bioaccumulative and highly toxic form MeHg. Fish concentrate both ionic Hg and MeHg through consumption of water and aquatic organisms. However, the half-life of MeHg in fish is approximately two years, compared to almost three months for ionic Hg [22]. This slow loss, combined with continuous exposure, results in substantial MeHg accumulation along the food chain. Dietary consumption of fish is the primary pathway of human and wildlife exposure to MeHg, a potent neurotoxin that can significantly impair human health [2]. More than 95% of the total Hg present in fish muscle tissue is MeHg [23]. Consequently, larger predatory fish generally have the highest MeHg concentrations [24]. Approximately 75% of total fish captures in the Peruvian Amazon are from subsistence fisheries; therefore, MeHg exposure in regions such as MDD is an important concern for riverine and indigenous populations that subsist on local fish [25].

Mercury concentrations are difficult to predict and track in aquatic ecosystems because of their complex interactions with multiple physical and biogeochemical factors that drive its transport, transformations and fate [26]. Diringer et al. [27] found higher Hg concentrations in fish (Hg_fish_) and sediment (Hg_sed_) at sites downstream from ASGM activities in comparison to upstream sites along the Madre de Dios River. Previous research in the study area have reported elevated Hg concentrations in human hair and in commercial fish species in locations with ASGM activities as well as in locations without ASGM activities [19,27,28,29,30,31,32]. Fish sampling in these rivers is difficult because waters tend to be uninhabitable by fish due to high turbidity from very high suspended solids loads [33].

In this study, we evaluated Hg_sed_ and Hg_fish_ in a river where mining activities were occurring upstream and compared these values to those from two proximate rivers without mining to characterize the level and extent of Hg contamination associated with ASGM. The objective of our study was to evaluate if proximity to ASGM is enriching Hg_sed_ and Hg_fish_ in the rivers. We also wanted to address two subsidiary questions: (1) How do Hg_fish_ vary by species, trophic position, and size? and (2) Do Hg_fish_ vary seasonally?

## 2. Materials and Methods

### 2.1. Site Description

**Figure 1:**
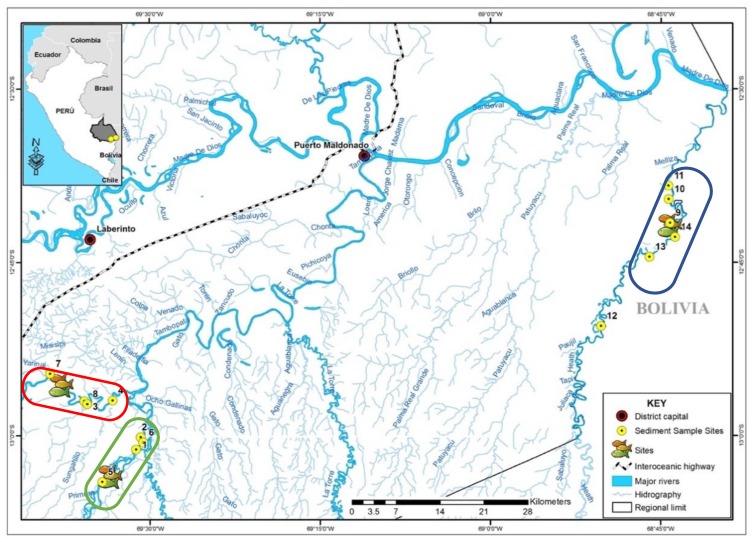
Map of the study region. Fish sampling for each season was conducted in the circled reaches for each river Tambopata River (green), Malinowski River (red), and Heath River (blue). Circles indicate sediment sample sites. The Interoceanic Highway is denoted with a dashed line.

MDD is located in the Amazon rainforest in southeast Peru bordering Brazil and Bolivia. Puerto Maldonado (12.5909° S, 69.1963° W) is the capital city of MDD. Madre de Dios, which directly translates as “Mother of God,” is known as the “biodiversity capital of Peru”. It is considered one of the most biodiverse ecosystems in the world and has been prioritized for conservation [34,35]. The construction of the Interoceanic Highway, a roadway which spans approximately 2600 km connecting Brazil and Peru (Figure 1, dashed line), has led to increased urbanization, industrialization, and natural resource extraction, all of which have degraded the environment [36]. In particular, 70,000 ha of virgin forest have been cleared for gold mining and an estimated 40–45 Mg of elemental Hg are used annually by miners to amalgamate gold flakes in excavated soil [14,16].

This study was conducted on three rivers in MDD. The Tambopata and Malinowski rivers are located southwest of the district capital, in the Tambopata National Reserve. Tambopata River receives inputs from the mining-impacted Malinowski River (Figure 1, circled in red). We sampled Tambopata River upstream from its confluence with Malinowski River (Figure 1, circled in green). Studies show that forest loss from ASGM on the upper Malinowski River has largely increased in the last decade [16]. The Heath River (circled in blue, Figure 1) is located southeast of the district capital in an undisturbed watershed within the Bahuaja Sonene National Park. This river forms the border between Peru and Bolivia. There have been no known mining activities in the national park up to the time of this study.

### 2.2. Sampling and Sample Processing Procedures

Wet season sampling was conducted in January–February 2017. Dry season sampling was conducted in August 2017. Wet season sampling was challenging because fish dispersed into floodplains and debris hindered boat travel. Nevertheless, fish and sediment samples were collected in both seasons. Sediment samples were collected using an Ekman dredge in quiescent pools with predominantly fine sediments. To complement river sediments, shoreline samples were also collected from recently submerged areas (following storm peaks) using a 2-cm deep, 10-cm wide stainless-steel scoop. All sediment collection equipment was rinsed thoroughly with deionized water between sample collections.

Fish samples were collected using drag nets, gill nets, and hook and line based on conditions at each site. Lengths (L, mm) and wet weight (WW, kg) were measured for each individual fish in the field. During wet season sampling, strong currents proved difficult for certain fishing techniques, particularly drag nets. During dry season sampling, with low-flow and low-water levels, all fishing techniques were viable. The majority of fish species caught were commercially important species, easily identified in the field. For less common fish species, identification was based on feeding habits and sampling location according to taxonomy keys [37,38]. For fish longer than 10 cm, a scalpel was used to remove the skin and scales from just below the dorsal fin of the left side of each fish. An 8-mm Acuderm biopsy punch (Acuderm Inc, Ft. Lauderdale, FL, USA) was then used to extract a small, subcutaneous “plug” of fish tissue for analysis. Several studies have shown that there is little variation between traditional, fish filet samples and fish plug samples [39,40,41,42]. For fish less than 10-cm long, a scalpel was used to remove the skin and a standard fillet sample was taken from the left side of the fish. All research protocols involving fish subjects were approved by Servicio Nacional de Áreas Naturales Protegidas por el Estado (Resolución Directoral N° 010-2017-SERNANP-DGANP).

We visited a total of 25 fish sampling sites: nine on Tambopata River, six on Malinowski River, and ten on Heath River (Supplementary Materials, Table S1). All fish sampling sites for Tambopata and Malinowski rivers were located upstream from their confluence. A total of 308 fish samples were collected during both seasons. During each cruise, fish and sediment samples were preserved on wet ice. Upon arrival in Puerto Maldonado, all samples were immediately stored at −20 °C. Samples were then transported with ice in a Crēdo ProMed^®^ cooler (Pelican BioThermal, Plymouth, MN, USA) to Syracuse University in Syracuse, NY for analyses.

Physicochemical water quality parameters (pH, temperature, dissolved oxygen, and nitrate) were measured *in-situ* at each site using a YSI Sonde (YSI Inc., Yellow Springs, OH, USA), Hanna pH Tester (Hanna Instruments, Woonsocket, RI, USA), and a LaMotte Dissolved Oxygen Test Kit (LaMotte Company, Chestertown, MD, USA). Turbidity was measured using a Hach 2100Q Portable Turbidimeter (Hach Company, Loveland, CO, USA).

### 2.3. Chemical Analyses

Upon arrival at Syracuse University, all samples were freeze-dried dried at −80 °C and 0.080 mBar using FreeZone: Type 6 plus freeze drier by Labconco (Labconco Corp., Kansas City, MO, USA). After freeze-drying, fish samples were homogenized and analyzed for total Hg using a Milestone Direct Mercury Analyzer (DMA-80, Milestone Inc., Shelton CT, USA) following US EPA Method 7473 [43]. Sediment samples were sieved and the fraction of particle sizes <63 µm were analyzed for total Hg (EPA Method 7473) [43] as well as organic carbon and total nitrogen using an elemental analyzer (EPA Method 440.0) (Costech, Valencia, CA, USA) [43,44]. Samples were weighed before and after freeze-drying in order to calculate percent moisture.

Duplicate measurements were taken for each fish and sediment sample analyzed for Hg, and the two results were averaged for the reported result. The average relative percent difference in total Hg measurements of duplicate samples was 4.5%. Quality control measures included testing of standard reference materials. Continuing calibration verification and continuing calibration blank measurements were determined on every tenth sample analyzed. The Method Detection Limit for total Hg analysis is 2 ng/g. No fish or sediment samples were rejected based on quality control results or duplicate relative percent differences.

Stable isotope analysis for fish tissue samples was completed at the University of California Davis Stable Isotope Facility using a PDZ Europa ANCA-GSL elemental analyzer interfaced to a PDZ Europa 20-20 isotope ratio mass spectrometer (Sercon Ltd., Cheshire, UK). Stable isotope composition is expressed in parts per thousand (‰ or ‘per mil’) as a deviation from a standard material. Nitrogen isotopic values were standardized against N_2_ gas in air as follows:

(1) δ15N (000)=[(RsampleRstandard)−1]×1000

where R = ^15^N/^14^N [45]. Similarly, carbon isotope samples (δ^13^C) were standardized against Vienna Pee Dee Belemnite, where R = ^13^C/^12^C. For every 11 field samples, one sample was randomly chosen for analysis as a lab duplicate.

Because there is considerable variation in δ^15^N at the base of the food web among ecosystems [46,47], we corrected δ^15^N values for each river using the average δ^15^N of *Prochilodus nigricans*, a primary consumer (e.g., a fish species that primarily feed on autochthonous algae and detritus). We calculated δ^15^N baseline-corrected trophic positions according to the following equation:

(2) $$\delta^{15}N\;\mathrm{baseline}\;\mathrm{corrected}\;\mathrm{trophic}\;\mathrm{position}=\;\frac{\delta^{15}N_{\mathrm{consumer}}\;-{\;\delta }^{15}N_{\mathrm{primary}\;\mathrm{consumer}}\;}{3.4}+2$$

where 3.4‰ represents the trophic fractionation [48,49].

### 2.4. Statistical Analyses

Three-way ANOVA without interaction terms was conducted to investigate the differences in physicochemical water quality parameters in the three rivers, between mainstem and tributary sites, and for two seasons. If there was a significant difference among rivers, then Tukey’s Honest Significant Difference (HSD) tests were used to perform pairwise comparisons among the three rivers.

Quantile–quantile plots were used to examine the normality of Hg_fish_ (Figure S1). Results suggested that log transformation would improve the normality of the dataset. Therefore, all statistical analyses were performed on log-transformed values. All Hg_sed_ reported were normalized to the average carbon content for comparison among sites.

Two-way ANOVA was conducted to investigate the differences in Hg_sed_ in the three rivers and two seasons. Two-way ANOVA was used to evaluate the differences in isotopic values (δ^15^N and δ^13^C) between the three rivers and two seasons. One-way ANOVA was used to examine any differences between mean log Hg_fish_ among the three rivers. Tukey’s HSD tests were used to perform pairwise comparisons among the three rivers. Due to the diversity of fish species being collected, fish size (length or mass) was not available as a covariate because of the wide ranges in fish species’ morphologies and growth rates did not provide a suitable correlation between size and Hg_fish_. Results from statistical analyses were considered significant when *p* < 0.05. *p*-values were adjusted for the multiple comparisons [50]. Analyses were performed in R 3.4.3 [51].

## 3. Results

### 3.1. Field Measurements and Physical Observations

Physicochemical water quality parameters were measured at each sampling site during both seasons. Values from sites in each river were averaged for mainstem and tributary sites separately (Table 1). Tributaries to Malinowski River were not measured for water quality during the dry season due to lack of flow.

Across all sites pH values were circum-neutral (6.3–7.6). Tambopata River had the highest mean (± standard deviation) pH values (7.4 ± 0.2) followed by Malinowski River (6.7 ± 0.4) and Heath River (6.7 ± 0.3). Tukey’s HSD showed Tambopata River had significantly higher pH values than both Malinowski and Heath rivers (*p* < 0.02). On average all temperature values were in the same range (26.4 ± 1.3 °C), but mainstem sites had significantly higher temperatures (27.1 ± 1.3 °C) than tributary sites (25.6 ± 1.1 °C, *p* = 0.041). Tambopata River had the highest mean dissolved oxygen (DO) concentrations (7.3 ± 0.2 mg/L), followed by Malinowski River (6.6 ± 0.3 mg/L) and Heath River (5.6 ± 1.6 mg/L). Tukey’s HSD identified a significant difference in DO concentrations among all rivers (Tambopata-Malinowski, *p* = 0.039; Malinowski-Heath, *p* = 0.003; Tambopata-Heath, *p* < 0.00001). Analysis showed significantly higher nitrate (NO_3_^−^) concentrations in the wet season (0.27 ± 0.09 mg N/L) compared to the dry season (0.11 ± 0.08 mg N/L, *p* = 0.0002), but not among rivers or tributaries vs. mainstem sites.

**Figure 2:**
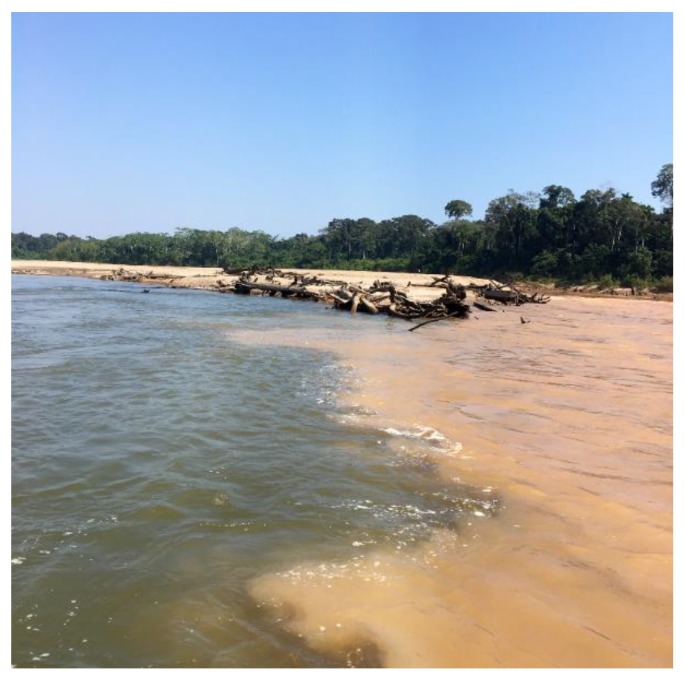
Photo looking upstream at the confluence of the relatively clear Tambopata River (left) and the more turbid Malinowski River (right), during the dry season sampling event in August 2017.

We noted that turbidity was significantly higher in the mainstem rivers (388 ± 272 NTU) than their tributaries (71 ± 77 NTU, *p* = 0.014) (Table 1). The single highest turbidity (890 NTU) was recorded at a mainstem site on Tambopata River during the wet season. The lowest turbidity (9 NTU) was recorded in a tributary to Malinowski River during the wet season. During the low-flow, dry season, the difference between the clear Tambopata (42 ± 31 NTU) and turbid Malinowski (401 ± 56 NTU) rivers was evident at their confluence (Figure 2). Despite the large difference in mean turbidity, only two sites measured in Malinowski River led to an adjusted *p*-value from Tukey’s HSD of 0.069. For Heath River, turbidity in the mainstem was higher during the dry season (644 ± 220 NTU) due to uncharacteristically large, daily precipitation events. However, in the wet season, Heath River had the lowest mean turbidity (305 ± 187 NTU) in mainstem sites in comparison to both Tambopata (581 ± 362 NTU) and Malinowski (359 ± 168 NTU) rivers.

### 3.2. Total Hg and % C in Sediment

Hg_sed_ were positively, linearly correlated with % C (R^2^ = 0.85) for all samples combined. Thus, Hg_sed_ were normalized to the average % C (0.39) using the linear relationship. % C values ranged from 0.03 to 0.98% (0.39 ± 0.29%). % C-normalized Hg_sed_ ranged from 13.9 to 367.2 μg/kg (21.3 ± 3.9 μg/kg). The highest Hg_sed_ was found immediately downstream from an ongoing ASGM site which was releasing mining tailings directly into Malinowski River. This value is considered an outlier, and therefore was excluded from the normalizing and statistical analyses.

**Figure 3:**
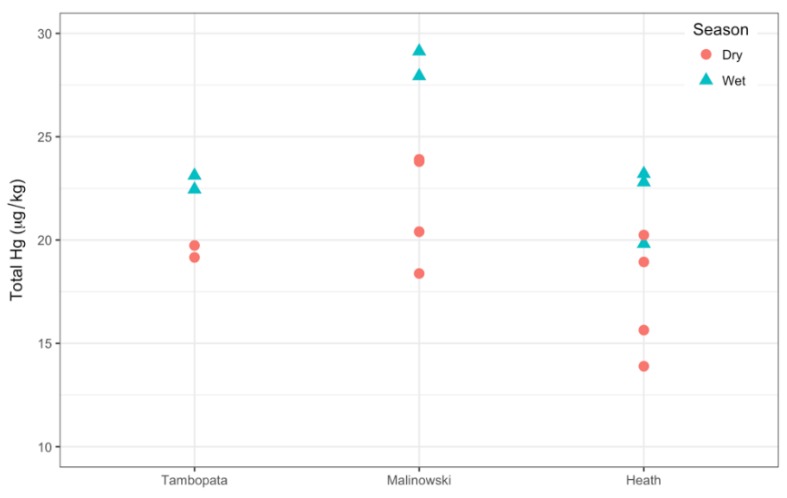
The % C-normalized Hg_sed_ (μg/kg) for each river. Dry season Hg_sed_ are shown with circles and wet season Hg_sed_ are shown with triangles. All wet season Hg_sed_ are higher than dry season Hg_sed_ for each river, except one value in Heath River. An outlier collected directly from an ASGM-site was not included (Malinowski River = 367.2 μg/kg).

The two highest Hg_sed_ values were measured in Malinowski River during the wet season (29.1 and 27.9 μg/kg) (Figure 3). The two lowest Hg_sed_ values were measured in samples from Heath River during the dry season (13.9 and 15.6 μg/kg). On average, Hg_sed_ were significantly higher in the wet season (24.1 ± 3.3 μg/kg) than in the dry season (19.4 ± 3.1 μg/kg, *p* < 0.001). The ASGM-impacted Malinowski River had the highest mean Hg_sed_ (23.9 ± 4.1 μg/kg), followed by Tambopata River (21.1 ± 1.9 μg/kg) and Heath River (19.2 ± 3.4 μg/kg). There was no evidence of interaction between river and season (*p* = 0.49), but there was a significant difference in Hg_sed_ among the three rivers (*p* = 0.01). There was a significant difference in Hg_sed_ between Malinowski and Heath rivers (*p* = 0.008), but no difference between other pairwise comparisons.

### 3.3. Hg in Fish

Hg_fish_ data indicated significant differences among the three rivers. Tukey’s HSD showed that Hg_fish_ in Malinowski River were significantly different than Tambopata (*p* = 0.02) and Heath (*p* = 0.0001) rivers. Various factors were explored (length, season, and baseline-corrected trophic position) to further investigate differences in Hg_fish_ after controlling for these factors.

#### 3.3.1. Fish Length

More than 35 species of fish were collected among all sites (Table S2), ranging in length from 66 mm to 980 mm (median 205 mm). Fish length is a common scaling factor for Hg_fish_; therefore, we attempted to normalize Hg_fish_ to median lengths for species for which >5 individuals were collected. However, Hg_fish_ were not strongly correlated with length. Therefore, we present unadjusted Hg_fish_. Hg_fish_ ranged from 0.01 mg/kg to 1.50 mg/kg (median 0.14 mg/kg). The highest Hg_fish_ was found in a piscivorous paña *Serrasalmus* spp. (1.50 mg/kg) sampled in the ASGM-impacted Malinowski River. The fish was only 79 mm long. The longest fish sampled was 980 mm, a piscivorous puma zungaro *Pseudoplatystoma tigrinum* (0.82 mg/kg) caught in Heath River.

#### 3.3.2. Wet vs. Dry Season

The mean Hg_fish_ in Malinowski River was significantly higher in the dry season (0.28 ± 0.24 mg/kg) than in the wet season (0.18 ± 0.15 mg/kg, *p* = 0.028) (Table 2). However, for Tambopata and Heath rivers mean Hg_fish_ were similar in the wet (Tambopata: 0.23 ± 0.23 mg/kg; Heath; 0.20 ± 0.22 mg/kg) and dry seasons (Tambopata: 0.22 ± 0.27 mg/kg, *p* = 0.508; Heath: 0.19 ± 0.27 mg/kg, *p* = 0.072). ANOVA among rivers and seasons showed there was no significant difference between seasons (*p* = 0.33). Therefore, subsequent comparisons are based on data combined for both seasons.

#### 3.3.3. Fish Isotopic Values (δ^15^N and δ^13^C)

The δ^15^N values ranged from 3.9 to 12.4‰ (9.4 ± 1.5‰) for the entire dataset. Mean δ^15^N values were lowest in Tambopata River (8.6 ± 1.4‰), followed by Heath River (9.6 ± 1.4‰) and Malinowski River (9.8 ± 1.4‰). Two-way ANOVA showed there was no significant difference between seasons (*p* = 0.82), but there was a significant difference among the three rivers (*p* < 0.000001). Tukey’s HSD indicated a significant difference in δ^15^N values between Tambopata and both Heath and Malinowski rivers (*p* < 0.00001).

The δ^13^C values ranged from −39.4 to −14.9‰ (−31.3 ± 3.9‰) for the entire dataset. Mean δ^13^C values were most depleted in Heath River (−33.2 ± 3.6‰), followed by Malinowski River (−29.8 ± 2.3‰) and Tambopata River (−28.4 ± 3.6‰). Two-way ANOVA showed there was no significant difference between seasons (*p* = 0.10), but there was a significant difference among the three rivers (*p* < 0.000001). Tukey’s HSD showed there was a significant difference in δ^13^C values among all rivers (Tambopata-Malinowski, *p* = 0.037; Malinowski-Heath, *p* < 0.000001; Tambopata-Heath, *p* < 0.000001).

Log-transformed Hg_fish_ were positively, linearly correlated with δ^15^N baseline corrected trophic position for each river: Tambopata River, R^2^ = 0.52; Malinowski River, R^2^ = 0.51; Heath River, R^2^ = 0.45. Thus, log Hg_fish_ were normalized to median δ^15^N baseline-corrected trophic position (2.3) for all fish samples, using the linear model constructed for each river. Once normalized, the highest mean Hg_fish_ were in Tambopata River (0.22 ± 0.14 mg/kg), followed by Malinowski River (0.17 ± 0.10 mg/kg), and lastly Heath River (0.16 ± 0.13 mg/kg). ANOVA showed significant differences in mean log Hg_fish_ among the three rivers (*p* = 0.03). Tukey’s HSD indicated there was a significant difference in mean log Hg_fish_ only between Tambopata and Heath rivers (*p* = 0.037).

Tambopata River had the highest fraction of fish (27%) above the United States Environmental Protection Agency fish tissue-based water quality criterion of 0.3 mg Hg/kg [52]. Heath River had the intermediate fraction of fish caught above the health consumption concentration criterion (13%), while Malinowski River had the lowest fraction of fish above the criterion (9%). Although the criterion is for MeHg, the USEPA suggests that 90 to 95 (or greater) percent of total Hg in fish tissue is MeHg. Therefore, we assumed the Hg_fish_ occurred as MeHg, and compare results directly to this criterion. Heath River had the most fish, 20 of (154), with Hg_fish_ above the health consumption criteria. Tambopata River and Malinowski River had 16 (of 61) fish and 7 (of 80) fish, respectively, above the criterion.

## 4. Discussion

Although the major waterways studied in MDD are generally similar to each other in terms of their physicochemical water quality conditions, environmental degradation was apparent in Malinowski River’s very turbid water indicative of upstream land disturbance. Hg_sed_ measured for this study are consistent with prior studies that evaluated Hg_sed_ from the ASGM-impacted Malinowski River [27,30,33]. Riverine sites closer to ASGM activities tended to have higher Hg_sed_ (i.e., Malinowski and Tambopata rivers), while rivers more remote from mining activities tended to have lower Hg_sed_ (i.e., Heath River). The highest Hg_sed_ measured directly downstream from an ASGM site on the Malinowski River was an order of magnitude higher than all other Hg_sed_ in this study, similar to concentrations measured in other studies near mining activity [10,53,54]. Hg_sed_ varied significantly between seasons, with higher Hg_sed_ during the wet season. These higher Hg_sed_ could be due to transport of Hg contaminated soil from denuded landscapes where mining occurs or the remobilization of river sediments from turbulence associated with runoff events [9,10]. This observation raises concern of the impacts of ASGM on Hg mobilization by sediment transport due to tailings from mining activities that are either directly released or washed into waterways.

In spite of the higher turbidity and Hg_sed_, we do not find that Hg contamination is strongly expressed as higher Hg_fish_ downstream of ASGM. The lack of a relationship between Hg_sed_ and Hg_fish_ is consistent with other regional Hg studies [55,56,57,58]. Gold mining along the Malinowski River, and elsewhere in MDD, remains localized relative to their large watersheds. Thus, local activities appear to have local impacts, but are not so large contaminate to the entire river downstream from mining sites. It is not clear how much gold mining is contributing to the somewhat elevated concentrations. Apparently, Hg supply is only one factor influencing Hg_fish_. Likely, complex interactions among multiple factors that influence the transport, bioavailability, methylation and trophic transfer of Hg contribute to Hg_fish_ in MDD.

Larger fish generally have higher Hg_fish_ than smaller fish of the same species [59,60]. However, Hg_fish_ in MDD rivers were not directly correlated with fish size, similar to results reported from other studies in the Amazon [9,27,61]. The tremendous diversity in habitat, feeding patterns and trophic structure in the Peruvian Amazon likely over-shadowed typical bioaccumulation processes for any particular specie [62]. Moreover, high species diversity among sampling sites precluded direct comparisons of any one specie. However, δ^15^N baseline-corrected trophic position were positively, linearly correlated with Hg_fish_ for each river. Suggesting that, similar to previous studies in the region, feeding behavior and trophic position are better bioindicators of Hg_fish_ than fish size in the region [27,33]. We found only one detritivorous fish, *yulilla*, with Hg_fish_ higher than the USEPA criterion. All other species above the criterion were omnivores or piscivores, i.e., *chambira, corvina, dentón, doncella, paña, pejeperro*, *pirillo, pico de pato*, *puma zungaro*, *raya*, and *toa*.

Even though the highest individual Hg_fish_ was observed in the Malinowski River, mean normalized Hg_fish_ from the Malinowski River were similar to Hg_fish_ in the other rivers. Unexpectedly, Malinowski (0.17 ± 0.10 mg/kg) and Heath (0.16 ± 0.13 mg/kg) rivers had similar mean normalized Hg_fish_. Tambopata River had the highest mean normalized Hg_fish_ (0.22 ± 0.14 mg/kg). There was not a seasonal difference in Hg_fish_, in contrast to the seasonal difference in Hg_sed_. Therefore, future studies should consider sampling in both wet and dry seasons to more fully capture any seasonal variation. Not portrayed in these Hg_fish_ results, however, are differences in aquatic habitats within a given river.

Mean δ^15^N in fish were lower in Tambopata River (8.6 ± 1.4‰), in comparison to both Heath (9.6 ± 1.4‰), and Malinowski rivers (9.8 ± 1.4‰). δ^15^N ratio is a proxy for the relative trophic position. δ^15^N becomes more enriched with each subsequent trophic position. Higher Hg_fish_ in Tambopata River were unexpected given their lower δ^15^N because lower trophic levels fish tend to have lower Hg concentrations [27,28,33,63]. Mean δ^13^C was most depleted in Heath River throughout both seasons (−33.2 ± 3.6‰), followed by Malinowski (−29.8 ± 2.3‰) and Tambopata (−28.4 ± 3.6‰) rivers. Although there were no statistical differences in isotopic values between seasons, differences were evident among rivers. δ^13^C ratio is a signature of the source of energy at the base of the food chain because it fractionates very little in biota. More depleted δ^13^C values are usually associated with photosynthetic C fixation [64]. The lighter δ^13^C in Heath River indicate a phytoplankton-driven ecosystem and fish eating on detritus material [65,66]. The more isotopically fractionated (more positive) δ^13^C ratios in fish from Tambopata and Malinowski rivers are most probably a result of fish feeding on carbon-limited benthic phytoplankton. The differences in the energy source to these ecosystems is probably driven by the different biogeochemistry. This linkage and potential influence on the fish Hg accumulation warrens a further study.

While fish in the ASGM-impacted Malinowski River did not exhibit particularly high Hg_fish_, mining activities still appear to negatively impact their aquatic ecosystem. Studies suggest that elevated turbidity, resulting from an increased load of suspended sediment such as observed in Malinowski River, leads to a reduction of fish diversity and shifts community structure [67]. The turbidity reduces light transmission, which in turn affects the aquatic food web by both reducing photosynthesis and limiting visual foraging efficiency of many fishes [68,69].

Similar to previous studies conducted in ASGM-impacted river reaches, we found it difficult to collect fish where ASGM was most intense [33,70]. Nonetheless, after spending 20+ hours in Malinowski River, we were able to collect 80 fish samples over both seasons. Sight-feeding detritivores made up only 7% of fish samples for Malinowski River compared to more than 50% of the fish samples in both Tambopata and Heath rivers. Lower trophic position fish, which consume detritus and plants, seem less successful in the turbid waters of Malinowski River with no submerged aquatic vegetation. Economically important fishes present in MDD fish markets (e.g., *bocachico, yahuarachi, doncella*) are apparently absent from Malinowski River [70].

ASGM-enhanced soil erosion plays an important role in altering the physical structure of habitats by limiting or eliminating food sources for certain fish species [69]. Future research of Hg_fish_ in this region should use carbon and nitrogen isotopes of both fish and primary food sources to investigate Hg_fish_ among fish species with similar feeding habits, as well as the effects of ASGM activities on aquatic food webs. Our findings suggest that further monitoring of Hg_fish_ from MDD could be important to evaluate exposure to Hg in these sensitive environments.

Fish in MDD can be a good indicator of Hg risk and exposure for nearby riverine communities as studies have shown that persons who consume more fish tend to have higher Hg in hair [19,32]. Mercury in human hair may not be the best indicator of Hg contamination at a given riverine site due to many extrinsic and intrinsic drivers which influence pathways of Hg bioaccumulation in food webs [57]. For example, Wyatt et al., (2017) found that the highest hair Hg in communities strongly connected to Madre de Dios River occurred upstream—but downwind—from active mining. For most organisms, exposure to MeHg mainly occurs through diet, whereas for humans associated with ASGM inorganic Hg exposure can also occur through inhalation [7,71].

In order to obtain a better understanding of Hg risk to wildlife and to avoid confounding factors such as migration and variation in trophic position, future studies might utilize juvenile fish, which typically provide a more site-specific measure of relative Hg exposure and uptake in comparison to larger, predatory fish [59,71,72].

## 5. Conclusions

Hg_fish_ results from this study are similar to results reported by others in the Amazon. Accounting for fish size, species and trophic position, Hg_fish_ were not found to be elevated in the ASGM-impacted Malinowski River in comparison to the other two rivers sampled. Higher Hg_fish_ were correlated with higher trophic position fish, predominantly piscivores, thus indicating that regardless of the proximity to ASGM activity, feeding behavior and food availability play an important role in Hg bioaccumulation. Future investigations in the region should focus on food web dynamics to elucidate effects caused by ASGM.

Most water quality parameters did not portray effects of ASGM; however, the increased turbidity indicated environmental degradation in the ASGM-impacted Malinowski River. Although, Hg loadings from ASGM activities did not translate to higher Hg_fish_, it did account for higher Hg_sed_. Sediment samples proved to be better indicators of Hg contamination for local river conditions. Our findings highlight the importance of annual sediment sampling for monitoring Hg contamination and transport along riverine sites.

Due to the high complexity of the Amazonian aquatic ecosystem studied, a direct comparison of Hg_fish_ among sites may not fully explain the variations in Hg contamination. Hg_fish_ concentrations are a good indicator of Hg exposure to riverine communities that rely heavily on fish as a source of food and protein; however, these values alone are not a full representation of ecosystem health. Many confounding variables need to be considered when comparing Hg_fish_ among different sites, such as: species, trophic position, feeding behavior, habitat degradation, and energy sources. Follow-up Hg studies in biota should include isotopic signatures from distinct seasons in order to examine the variation in food availability and energy sources.

## Figures and Tables

**Table 1:** Average physicochemical water quality parameters gathered from mainstem (M) and tributary (T) sites from each river for both wet and dry seasons. Values presented are means (± standard deviation).

River	Season	Site (M/T)	pH	Temperature (°C)	Dissolved Oxygen (mg/L)	NO_3_^−^ (mg N/L)	Turbidity (NTU)
Tambopata	Wet	M	7.5	26.3 (0.41)	7.4 (0.04)	0.29 (0.06)	581 (362)
	Wet	T	7.6	27.5	7.0	0.26	215
	Dry	M	7.4 (0.23)	25.7 (0.28)	7.6 (0.17)	0.05 (0.03)	42 (31)
	Dry	T	7.3	24.6	7.4	0.03	12
Malinowski	Wet	M	7.1 (0.42)	28.1 (0.55)	6.9 (0.17)	0.25 (0.14)	359 (169)
	Wet	T	6.3	24.8	6.7	0.12	9
	Dry	M	6.9 (0.02)	28.9 (1.6)	6.3 (0.36)	0.09	401 (56)
	Dry	T	NS ^1^	NS	NS	NS	NS
Heath	Wet	M	6.5 (0.40)	26.1 (1.1)	5.0 (0.33)	0.28 (0.12)	305 (188)
	Wet	T	6.4 (0.02)	25.1 (1.3)	4.9	0.34 (0.03)	79 (18)
	Dry	M	7.2 (0.04)	27.5 (1.9)	6.2 (0.84)	0.23 (0.02)	644 (221)
	Dry	T	6.6	25.9	6.2	0.06	33

^1^ NS (no sample) was collected or measurement taken due to tributaries being dry.

**Table 2:** Mean fish Hg concentrations (±standard deviation) for each season.

River	Season	Mean Hg_fish_ (mg/kg)
Tambopata	Wet	0.23 ± 0.23
	Dry	0.22 ± 0.27
Malinowski	Wet	0.18 ± 0.15
	Dry	0.28 ± 0.24
Heath	Wet	0.20 ± 0.22
	Dry	0.19 ± 0.27

## Supplementary Materials

The following are available online at http://www.mdpi.com/1660-4601/15/8/1584/s1, Table S1: Sample site list with Global Positioning System coordinates. Sites 1-9 correspond to Tambopata River, sites 10-15 correspond to Malinowski River, and sites 16–25 correspond to Heath River. Sites with * were also sampled for sediment, Figure S1: Quantile–quantile plots examining normality of Hg_fish_ data: (a) raw Hg_fish_ data and (b) log-transformed Hg_fish_ data, Table S2: Raw mercury concentrations for fish samples collected during both wet and dry season sampling events for all rivers combined. Sampling Location identifies where each specie was collected—Tambopata River (T), Malinowski River (M), and Heath River (H).
