# Supplementary material for: Mercury Contamination in Riverine Sediments and Fish Associated with Artisanal and Small-Scale Gold Mining in Madre de Dios, Peru
Source: Int J Environ Res Public Health. 2018 Jul 26;15(8):1584. doi: 10.3390/ijerph15081584 (PMC6121527; doi:10.3390/ijerph15081584)
Supplement: Supplementary file 1 [file ijerph-15-01584-s001.pdf]

Supplementary Materials:

Table S1. Sample site list with Global Positioning System coordinates. Sites 1-9 correspond to Tambopata River, sites 10-15 correspond to Malinowski River, and sites 16-25 correspond to Heath River. Sites with * were also sampled for sediment.

Site Name	Latitude	Longitude
1 – Chuncho* (T)	13° 1’ 12”	69° 31’ 13”
2 – Chuncho 2* (T)	13° 0’ 9”	69° 30’ 49”
3 – Colpa Chuncho (T)	12° 59’ 1”	69° 29’ 49”
4 – 8 Gallinas 1 (T)	12° 57’ 59”	69° 29’ 50”
5 – 8 Gallinas 2 (T)	12° 57’ 33”	69° 29’ 52”
6 – 8 Gallinas 3* (T)	13° 4’ 0”	69° 34’ 13”
7 – Isla de Monos* (T)	13° 0’ 36”	69° 30’ 34”
8 – Isla de Monos 2 (T)	12° 57’ 47”	69° 30’ 45”
9 – Boca Alto Tambopata (T)	12° 57’ 11”	69° 31’ 38”
10 – Alto Tetas Malinowski (M)	12° 56’ 41”	69° 34’ 29”
11 – Aguas Negras Alto Malinowski* (M)	12° 57’ 16”	69° 35’ 31”
12 – Alto Malinowski (M)	12° 56’ 45”	69° 34’ 42”
13 – Bajo Malinowski* (M)	12° 56’ 57”	69° 33’ 17”
14 – Quebrada Yarinal* (M)	12° 54’ 37”	69° 38’ 47”
15 – Tambopata Boca Malinowski (M)	12° 54’ 9”	69° 31’ 6”
16 – Lago Guacamayo 1* (H)	12° 41’ 36”	68° 44’ 10”
17 – Lago Guacamayo 2 (H)	12° 41’ 28”	68° 43’ 48”
18 – Lago Guacamayo 3 (H)	12° 41’ 49”	68° 44’ 10”
19 – Quebrada Maquisapa* (H)	12° 39’ 60”	68° 43’ 52”
20 – Quebrada San Antonio* (H)	12° 39’ 32”	68° 44’ 17”
21 – Quebrada Dario (H)	12° 38’ 22”	68° 44’ 18”
22 – Alto Heath* (H)	12° 50’ 32”	68° 50’ 15”
23 – Lago Heath (H)	12° 48’ 26”	68° 50’ 5”
24 – Grande Piedra* (H)	12° 44’ 32”	68° 45’ 58”
25 – Quebrada Boli* (H)	12° 42’ 49”	68° 43’ 43”

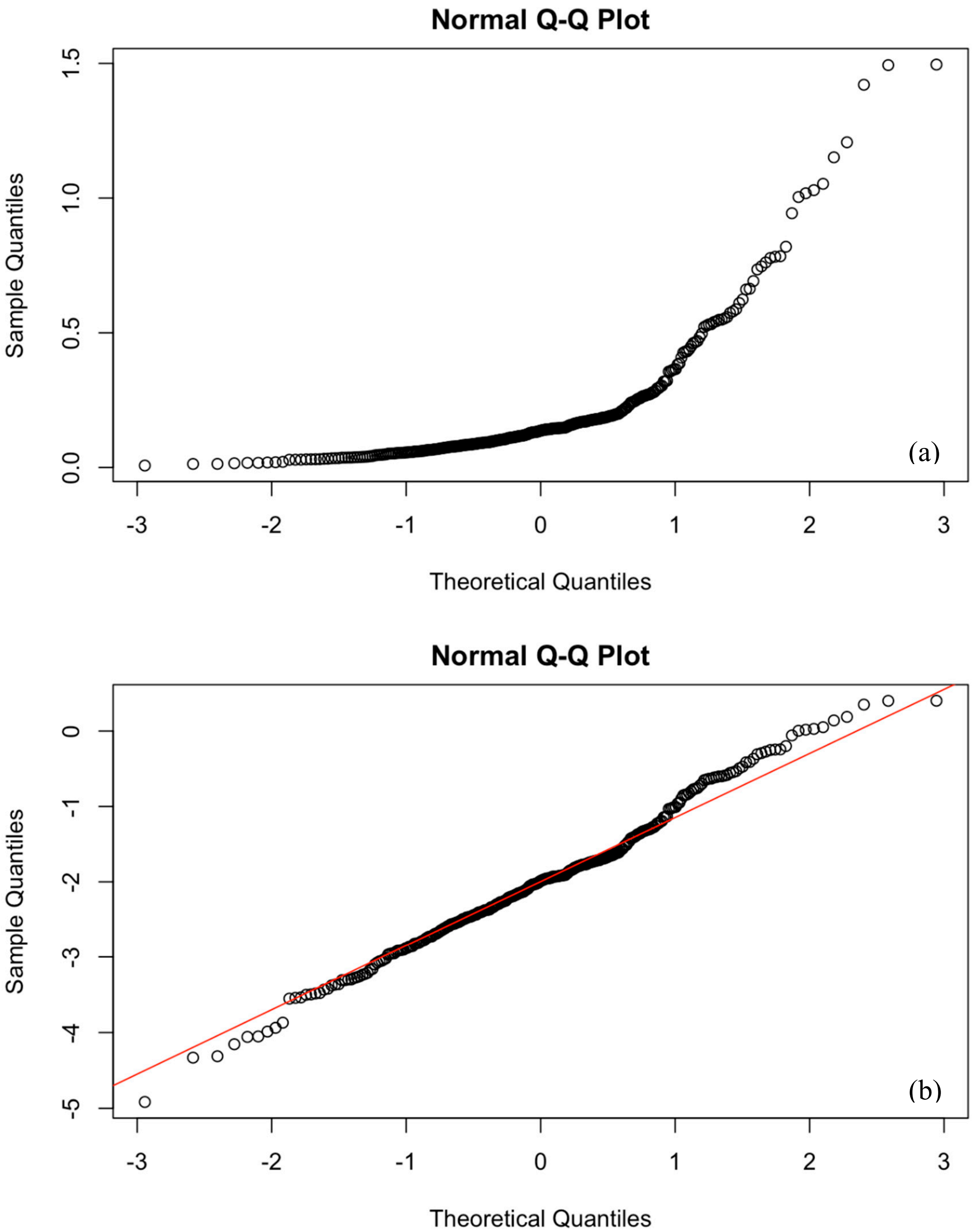

Figure S1. Quantile-quantile plots examining normality of Hg_{fish} data: (a) raw Hg_{fish} data and (b) log-transformed Hg_{fish} data.

Table S2. Raw mercury concentrations for fish samples collected during both wet and dry season sampling events for all rivers combined. Sampling Location identifies where each specie was collected—Tambopata River (T), Malinowski River (M), and Heath River (H).

Common Name	Species	Samples (n=308)	Sampling Location[1]	T-Hg (mg/kg) mean (Min, Max)	Strd. Dev. (mg/kg)	Mean Length (mm)	Mean Weight (g)
Añashua	*Crenicichla semicincta*	1	T	0.05	--	80	8
Anguila	*Electrophorus electricus*	1	T	0.50	--	720	1520
Bagre	*Exallodontus agaunai, Megalonema platycephalum, Pimelodus sp.*	21	T, M, H	0.13 (0.03, 0.24)	0.06	182 (118, 236)	139 (24, 260)
Bocachico	*Prochilodus nigricans*	39	T, M, H	0.10 (0.02, 0.28)	0.06	301 (208, 380)	722 (260, 1460)
Bocon, Toa	*Ageniosus inermis*	3	H	0.60 (0.47, 0.74)	0.14	377 (325, 411)	953 (620, 1140)
Bujurqui	*Satanoperca jurupari*	18	M	0.14 (0.09, 0.21)	0.04	114 (104, 134)	49 (35, 77)
Canero	*Cetopsis coecutiens*	2	T, M	0.32 (0.17, 0.47)	0.21	120 (77, 164)	64 (8, 120)
Carachama	*Hypostomus sp, Lamontichthys filamentosus, Squaliforma emarginata*	24	T, M, H	0.04 (0.02, 0.08)	0.02	190 (69, 265)	162 (8, 338)
Chambira	*Cynodon gibbus*	7	T, H	0.72 (0.12, 1.20)	0.41	248 (151, 363)	214 (80, 390)
Chio-chio	*Psectrogaster rutiloides*	1	H	0.04	--	158	140
Corvina	*Pachypops fourcroi*	1	T	0.43	--	196	130
Dentón	*Charax sp, Cynopotamus amazonus, Galleocharax sp, Roeboides affinis*	7	T	0.40 (0.16, 0.61)	0.18	156 (79, 228)	67 (7, 160)
Doncella	*Pseudoplatystoma tigrinum*	2	T, H	0.86 (0.78, 0.94)	0.12	641 (606, 676)	3595 (3360, 3830)
Gamitana	*Colossoma macropomum*	1	H	0.16	--	432	3320
Huasaco	*Hoplias malabaricus*	6	T, M	0.15 (0.10, 0.22)	0.04	143 (77, 205)	71 (7, 160)
Lamista	*Tetragonopterus argenteus*	1	T	0.09	--	112	70

Lisa	*Leporinus friderici*	4	H	0.11 (0.08, 0.14)	0.04	255 (240, 271)	348 (260, 430)
Macana	*Eigenmannia virescens, Sternarchorhynchus sp*	6	M	0.14 (0.07, 0.20)	0.05	192 (151, 232)	12 (7, 15)
Mota Punteada	*Calophysus macropterus*	1	M	0.29	--	399	540
Palometa	*Mylossoma aureum, Mylossoma duriventre, Myleus sp*	10	M, H	0.05 (0.01, 0.09)	0.03	154 (105, 217)	169 (43, 390)
Paña	*Pristobrycon sp, Serrasalmus cf compressus, Serrasalmus rhombeus, Serrasalmus spilopleuras*	31	M, H	0.53 (0.08, 1.50)	0.38	136 (75, 280)	145 (8, 830)
Pejeperro	*Acestrorhynchus falcatus*	7	M	0.34 (0.23, 0.44)	0.07	125 (104, 150)	28 (12, 47)
Pico de Pato	*Hemisorubim platyrhynchos, Platystomatichythys sturio, Sorubim lima*	9	T, M, H	0.53 (0.13, 1.15)	0.38	225 (91, 442)	180 (4, 630)
Pirillo	*Leptodoras acipenserinus, Nemadoras humeralis, Trachydoras steindachneri*	5	T, H	0.31 (0.20, 0.45)	0.10	168 (91, 215)	76 (11, 105)
Piro	*Megalodoras uranoscopus*	3	H	0.02 (0.01, 0.02)	0.002	382 (333, 418)	1737 (1060, 2240)
Platanito	*Auchenipterichthys sp*	2	M	0.11 (0.10, 0.11)	0.001	93 (90, 95)	9 (9, 10)
Puma Zungaro	*Pseudoplatystoma tigrinum*	1	H	0.82	--	980	10500
Raya	*Potamotrygon falkneri, Potamotrygon sp*	3	T, M	0.56 (0.53, 0.59)	0.03	636 (540, 697)	2227 (1450, 3050)
Sábalo	*Brycon amazonicus*	4	T, H	0.14 (0.05, 0.28)	0.10	391 (350, 474)	1445 (840, 2880)
Sapamama	*Triportheus albus, Triportheus angulatus*	10	T, H	0.14 (0.07, 0.29)	0.08	171 (148, 193)	104 (47, 160)
Sapocunchi	*Trachelyopterus cf galeatus*	1	H	0.10	--	168	150
Sardina	*Astyanax abramis, Astyanax bimaculatus,*	6	M	0.09 (0.05, 0.19)	0.05	82 (66, 104)	15 (9, 29)

	Astyanax maximus						
Tucunare	*Cichla monoculus*	1	H	0.19	--	203	210
Turushuki	*Oxydoras niger*	1	H	0.25	--	540	2440
Yahuarachi	*Potamorhina altamazonica*	64	H	0.12 (0.03, 0.026)	0.05	215 (165, 289)	268 (90, 620)
Yulilla	*Anodus elongatus, Hemiodus microlepis*	4	T, H	0.28 (0.06, 0.56)	0.21	262 (204, 294)	298 (250, 340)

[1] Tambopata River (T), Malinowski River (M), and Heath River (H).
